# Interleukin-7 Regulates Adipose Tissue Mass and Insulin Sensitivity in High-Fat Diet-Fed Mice through Lymphocyte-Dependent and Independent Mechanisms

**DOI:** 10.1371/journal.pone.0040351

**Published:** 2012-06-29

**Authors:** Stéphanie Lucas, Solenne Taront, Christophe Magnan, Laurence Fauconnier, Myriam Delacre, Laurence Macia, Anne Delanoye, Claudie Verwaerde, Corentin Spriet, Pasquine Saule, Gautier Goormachtigh, Laurent Héliot, Alain Ktorza, Jamileh Movassat, Renata Polakowska, Claude Auriault, Odile Poulain-Godefroy, James Di Santo, Philippe Froguel, Isabelle Wolowczuk

**Affiliations:** 1 Laboratory of Neuroimmunoendocrinology, Institut Fédératif de Recherche (IFR) 142, Univ Lille Nord de France, Institut Pasteur, Lille, France; 2 Laboratory of Genomics and Metabolic Diseases, Centre National de la Recherche Scientifique (CNRS) Unité Mixte de Recherche (UMR) 8199, Univ Lille Nord de France, Institut Pasteur, Lille, France; 3 Laboratory of Physiopathology of Nutrition, CNRS UMR 7059, Paris 7 Univ, Paris, France; 4 Laboratory of Cellular Immunopathology of Infectious Diseases, CNRS UMR 8527, Institut de Biologie/Institut Pasteur, Lille, France; 5 Interdisciplinary Research Institute, CNRS Unité de Service et de Recherche (USR) 3078, Univ Lille Nord de France, Villeneuve d’Ascq, France; 6 Laboratory of Biology and Pathology of the Endocrine Pancreas, CNRS Equipe d’Accueil Conventionnée (EAC) 4413, Paris 7 Univ, Paris, France; 7 Inserm U837 and Jean-Pierre Aubert Research Center, Centre Hospitalier Universitaire (CHU), Univ Lille Nord de France, Lille, France; 8 Innate Immunity Unit, Institut Pasteur, Paris, France; 9 Inserm U668, Paris, France; Boston University, United States of America

## Abstract

Although interleukin (IL)-7 is mostly known as a key regulator of lymphocyte homeostasis, we recently demonstrated that it also contributes to body weight regulation through a hypothalamic control. Previous studies have shown that IL-7 is produced by the human obese white adipose tissue (WAT) yet its potential role on WAT development and function in obesity remains unknown. Here, we first show that transgenic mice overexpressing IL-7 have reduced adipose tissue mass associated with glucose and insulin resistance. Moreover, in the high-fat diet (HFD)-induced obesity model, a single administration of IL-7 to C57BL/6 mice is sufficient to prevent HFD-induced WAT mass increase and glucose intolerance. This metabolic protective effect is accompanied by a significant decreased inflammation in WAT. In lymphocyte-deficient HFD-fed SCID mice, IL-7 injection still protects from WAT mass gain. However, IL-7-triggered resistance against WAT inflammation and glucose intolerance is lost in SCID mice. These results suggest that IL-7 regulates adipose tissue mass through a lymphocyte-independent mechanism while its protective role on glucose homeostasis would be relayed by immune cells that participate to WAT inflammation. Our observations establish a key role for IL-7 in the complex mechanisms by which immune mediators modulate metabolic functions.

## Introduction

Paradoxically, the disproportionate lack of white adipose tissue (WAT) in lipodystrophy and the excessive gain of WAT in obesity are both frequently associated with severe insulin resistance [1], [2]. This highlights the central role for adipose tissue as an essential organ for proper metabolic regulation.

Dysfunctional adipose tissue could participate in metabolic disease development notably through abnormal secretion of adipokines which have been shown to play important roles in insulin action, energy balance and inflammation [3]. Adipokines are considered as key players in the orchestration of the adaptive metabolic changes which occur at different cell and tissue levels. Indeed, as exemplified by leptin, adipokines can exert endocrine action on hypothalamic cells involved in food intake and/or energy expenditure [4]. Other factors, like IL-6 or TNFα, regulate adipose tissue mass and function, and modulate insulin sensitivity [5], [6]. Therefore, altered adipokine secretion associated to either excess or markedly reduced WAT mass may worsen the progression of the metabolic disease and promote insulin resistance, contributing to the development of type 2 diabetes [1]. Recently, immune-derived cytokines have also been shown to regulate WAT mass and function. Mice deficient in IL-18 show increased WAT mass and insulin resistance [7], while IL-15 overexpressing mice present reduced WAT mass [8]. IL-7 has recently been identified as a new adipokine whose expression and secretion are increased in the obese human adipose tissue [9]. Whether this endogenous production of IL-7 has any physiological function in adipose tissue and metabolism is still unknown.

IL-7 is a constitutively secreted cytokine primarily produced in bone marrow and peripheral lymphoid organs [10]. IL-7 is critically required for lymphocyte development and homeostasis [11], as first appreciated from the severe lymphopenia observed in IL-7 knockout and IL-7R knockout mice [12], [13], and later on in comparable immune deficiencies in humans who lack either IL-7 or components of its receptor [14], [15]. The key role of IL-7 in lymphocyte homeostasis was shown to rely on its control of basal lymphocyte glucose metabolism [16], maintaining high glucose uptake and expression of GLUT1 therefore allowing adequate glycolytic flux [17], [18], [19]. Beside its primary function in the regulation of the activation, growth and survival of lymphoid cells, it has been suggested that IL-7 can also act on non-lymphoid cells. Indeed, IL-7 has been shown to induce the production of pro-inflammatory IL-1, IL-6, IL-8 and TNFα by monocytes [20], to promote eosinophil and hippocampal neuron survival [21], [22] and to regulate osteoclast formation [23]. We recently extended IL-7 pleiotropic effects to energy balance regulation; indeed we reported that IL-7 directly targets the hypothalamic arcuate nucleus and protects mice from the development of monosodium glutamate-induced obesity [24].

Here we further assessed the effects of IL-7 on metabolism, focusing on the white adipose tissue. First, we characterized glucose homeostasis and WAT development in IL-7 transgenic mice (referred to as Tg IL-7, [25]). The reduced adipose tissue mass and the impaired glucose homeostasis of IL-7 overexpressing mice suggested that IL-7 might be a key factor in adipose tissue biology, as it was suspected from its increased expression in the obese human tissue [9]. Then, we aimed to analyze whether IL-7 metabolic effects revealed by its constitutive overexpression in mice could be extended to a more physiopathological context. We thus analyzed the consequences of acute IL-7 administration on fat mass accumulation, glucose intolerance and WAT inflammation in the high-fat diet (HFD)-induced obesity model in C57BL/6 mice. Finally, to distinguish between the lymphocyte-dependent and -independent metabolic action mechanisms of IL-7, the same parameters were analyzed in IL-7-treated HFD-fed severe combined immunodeficient (SCID) animals.

## Results

### White Adipose Tissue Mass and Sensitivity to Insulin are Impaired in IL-7 Overexpressing Mice

As a first step to study whether IL-7 might impact on the adipose tissue, we used IL-7 transgenic mice, in which overexpression is driven by the human keratin-14 (K14) promoter (Tg IL-7; [25]). IL-7 transgenic mice were fertile and healthy, slightly leaner than littermate wild-type controls (WT) (22.8±1.4 g and 25±2.9 g, respectively; *p* value = 0.12) yet presented similar BMI (0.28 g/cm^2^) and comparable consumption of standard diet.

**Figure 1:**
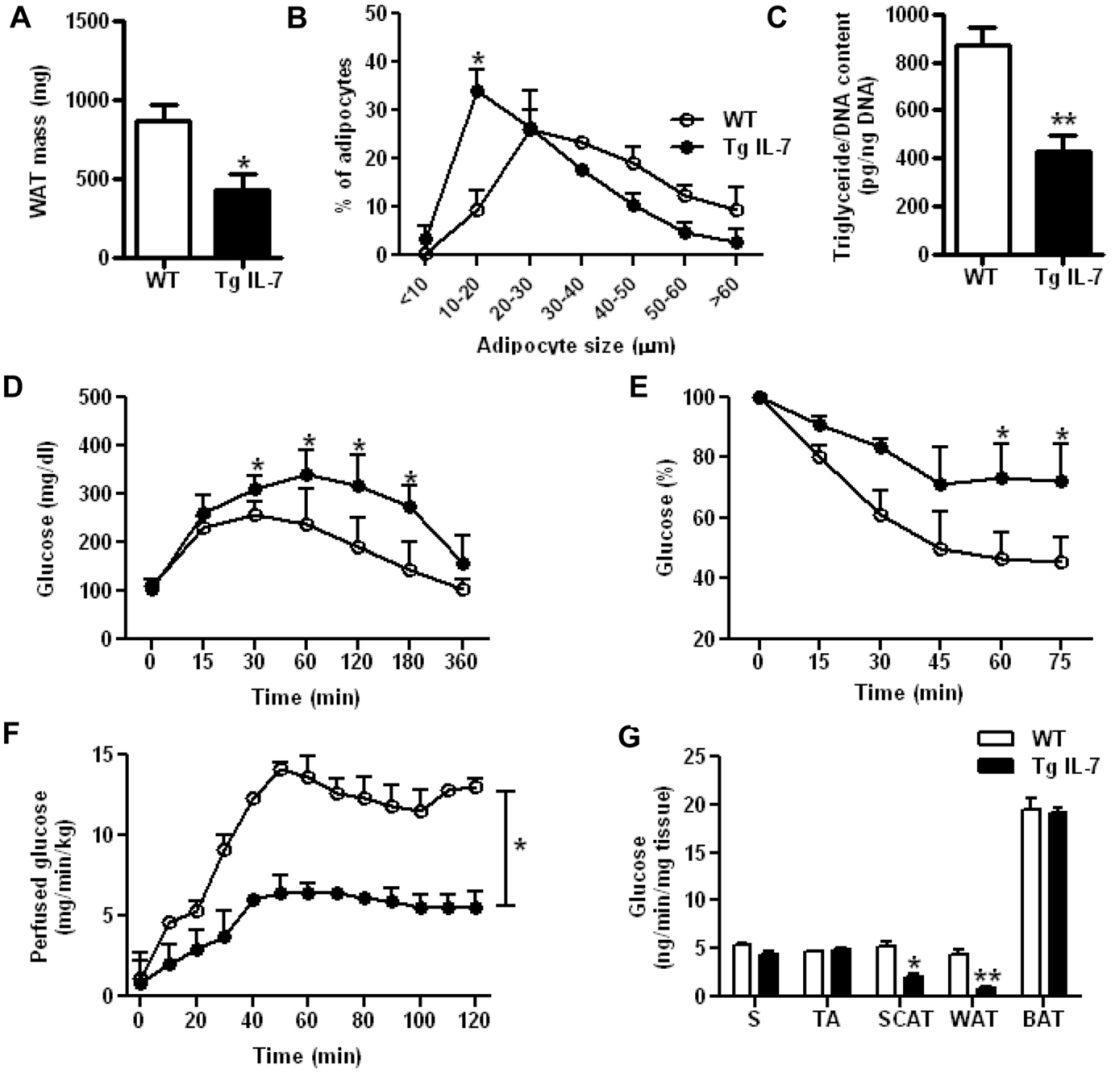
Reduced white adipose tissue mass and insulin sensitivity in IL-7 overexpressing mice. (**A**) Perigonadal WAT mass in transgenic mice and control animals. Results are expressed as mean ± SEM of 7 WT mice (white bars) and 8 Tg IL-7 mice (black bars). (**B**) Adipocyte diameter distribution of 3 WT (white circles) and 3 Tg IL-7 (black circles). (**C**) Ratio of total triglycerides to total DNA (in pg/ng). Results are expressed as mean ± SEM of 3 WT mice (white bars) and 5 Tg IL-7 mice (black bars). (**D**) Glucose tolerance test: Blood samples for glucose level determination were taken from each individual animal. Results are expressed as mean ± SEM of 5 WT (white circles) and 5 Tg IL-7 (black circles). (**E**) Insulin tolerance test: Glycaemia were individually measured at the indicated times. Data are presented as mean percentage of basal glycemia (t = 0 min) from average value ± SEM from 5 WT (white circles) and 5 Tg IL-7 (black circles). (**F & G**) *In vivo* hyperinsulinaemic-euglycaemic clamps were performed using [3-^3^H] glucose and 2-deoxy-D-[1-^14^C] glucose (2DG) for the estimation of whole body glucose fluxes (**F**) and tissue glucose uptake (**G**), respectively. The uptake of glucose was determined in one red fiber-type of muscle (*Soleus*, S), one white fiber-type of muscle (*Tibialis anterior*, TA), inguinal SCAT, perigonadal WAT, and brown adipose tissue (BAT). Results are expressed as the mean ± SEM of 5 WT (white bars) and 5 Tg IL-7 (black bars). Statistically significant differences between the groups are indicated as ^*^ *p*<0.05 and ^**^ *p*<0.01.

Compared with WT animals, standard diet-fed Tg IL-7 mice showed a 50% decrease in perigonadal WAT mass (Figure 1A), whereas brown adipose tissue, liver and spleen showed no macroscopical differences (data not shown). Histological examination of WAT sections showed a higher density of smaller adipocytes in Tg IL-7 mice (data not shown). Indeed, as shown in Figure 1B, morphometric analysis confirmed that Tg IL-7 adipocytes were smaller than WT adipocytes (64% adipocytes with a diameter <30 µm for Tg IL-7 mice *vs* 36% for WT mice; *p*<0.05^*^). In addition, adipocyte number per microscopical field was ∼3.5-fold higher in Tg than in WT mice (1,508±306 cells/mm^2^ for Tg IL-7 *vs* 426±35 cells/mm^2^ for WT, respectively; *p* value = 0.028^*^). The decrease in adipocyte size was also reflected by lower triglyceride content in the transgenic adipose tissue (Figure 1C). The DNA content per mg of tissue was higher for transgenic WAT compared to WT tissue (0.34±0.042 *vs* 0.21±0.025 µg/mg, respectively; *p*<0.05^*^). Since the mass of the tissue was reduced in transgenic animals compared to WT controls (Figure 1A), the resulting DNA content per fat pad is comparable between transgenic and control mice. Therefore, the decreased adiposity in Tg IL-7 mice is primarily due to decreased adipocyte size with no change in cellularity.

IL-7 overexpression was associated with increased levels of circulating free fatty acids (FFAs) and decreased levels of circulating triglycerides with no change in basal insulinemia and glycemia (Supporting Information, Table S1). Plasma FFAs concentrations are primarily governed by lipolysis in adipocytes and elevated FFAs levels are thought to restrict glucose utilization and induce insulin resistance. Indeed, *in vivo* glucose clearance was severely delayed in Tg IL-7 animals compared with WT mice, indicating glucose intolerance (Figure 1D). In addition, transgenic mice are insulin resistant since their hypoglycaemic response to insulin was lower compared to WT mice (Figure 1E). Hyperinsulinaemic-euglycaemic clamp experiments in both IL-7 overexpressing and control animals confirmed IL-7-induced insulin resistance: the glucose infusion rate needed to maintain euglycemia in the presence of a constant infusion of insulin was 2-fold lower in Tg IL-7 mice compared to control mice (13.7±0.5 mg/min/kg and 20.5±0.8 mg/min/kg for Tg IL-7 and WT, respectively; *p* value = 0.03^*^) (Figure 1F). In contrast, both groups exhibited similar hepatic glucose output (8.4±0.6 mg/min/kg and 8.7±1 mg/min/kg for Tg IL-7 and WT, respectively) suggesting that liver insulin sensitivity is not altered in Tg IL-7 mice. Then, we assessed the insulin-stimulated uptake of 2-deoxy-D glucose (2DG; a non-metabolized glucose analogue) in skeletal muscle (*soleus*, a predominantly red fiber-type muscle, and *tibialis anterior*, a predominantly white fiber-type muscle) and in brown and white adipose tissues (subcutaneous and perigonadal fat, respectively; SCAT and WAT). Although 2DG uptake was similar in muscle and brown fat tissue in both groups, the 2DG uptake in WAT was markedly lower in Tg IL-7 mice compared to WT mice (Figure 1G). IL-7 overexpression appeared to affect more perigonadal WAT than inguinal SCAT (respective decreases in 2DG uptake; 80% *vs* 60%), yet the expression levels of IL-7 have not been compared between WAT and SCAT. Altogether, these data support that the glucose uptake is severely perturbed in the two main white fat depots in IL-7 overexpressing mice.

### IL-7 Receptor is Mostly Expressed in the Stromal Vascular Fraction of C57BL/6 WAT

**Figure 2:**
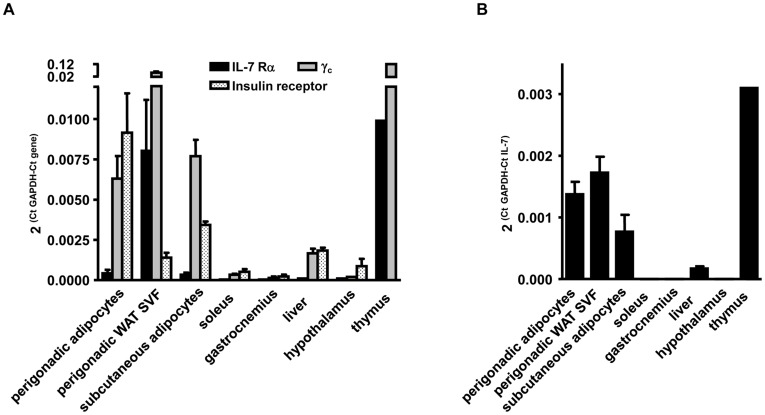
The IL-7 receptor is mostly expressed in the stromal vascular fraction of C57BL/6 adipose tissue. Expression levels of the mRNA of the IL-7 receptor subunits (IL-7Rα; black bars, γ_c_; grey bars) (**A**) and IL-7 (**B**) in different insulin sensitive tissues and in adipose tissue fractions isolated from three C57BL/6 male mice, using quantitative PCR. The insulin receptor mRNA levels are shown as control (spotted bars). Thymus was used as a positive control for the expression of IL-7 and IL-7R.

IL-7 activates target cells through its heterodimeric receptor (IL-7R; containing CD127 (IL-7Rα chain) coupled with CD132 (common γ chain: γ_c_)) [26]. To further characterize the expression level of IL-7 and IL-7R chains in insulin-sensitive tissues, real-time quantitative PCR analysis was performed on the adipocyte fraction and the stromal vascular fraction (SVF) isolated from the perigonadal WAT of C57BL/6 mice, and compared to the expression in skeletal muscles, liver and hypothalamus. It showed that IL-7 and IL-7Rα were expressed at higher levels in both WAT cell fractions than in the other tested metabolic tissues (Figure 2). Moreover, whereas IL-7Rα mRNA was mostly found expressed in the SVF which contains immune, endothelial and progenitor cells [27], the γ_c_ chain was well-detected in both fractions, yet at a higher level in the SVF (Figure 2A). IL-7 mRNA was equally expressed in the SVF fraction and the adipocyte fraction (Figure 2B).

These data suggest that WAT could be both a source and a target of IL-7 and strengthen that IL-7 could participate in the regulation of WAT functionality in metabolic diseases.

### A Single Administration of IL-7 Protects C57BL/6 Mice from High-fat Diet-induced WAT Mass Increase and Glucose Intolerance

**Figure 3:**
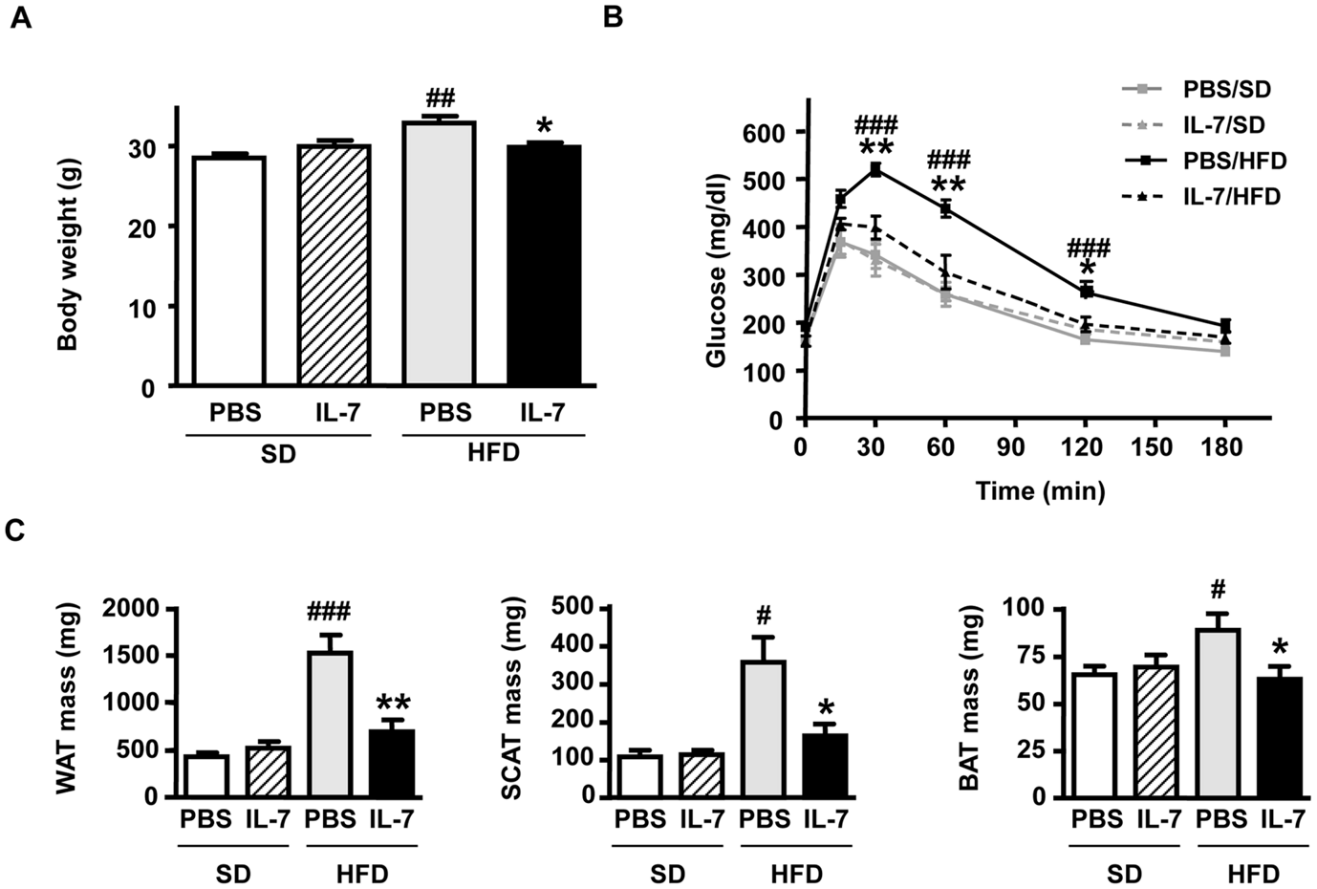
A single injection of IL-7 protects high-fat diet-fed C576BL/6 mice from obesity and glucose intolerance. (**A**) Body weight at 16 weeks of diet feeding (white bars; PBS/SD, hatched bars; IL-7/SD, grey bars; PBS/HFD, black bars; IL-7/HFD); (**B**) An i.p. glucose tolerance test was performed after 5 weeks of diet feeding (grey squares-continuous line; PBS/SD, grey triangles-dotted line; IL-7/SD, black squares-continuous line; PBS/HFD, black triangles-dotted line; IL-7/HFD); (**C**) Masses of perigonadal WAT, inguinal SCAT and interscapular BAT at 16 weeks of diet feeding (white bars; PBS/SD, hatched bars; IL-7/SD, grey bars; PBS/HFD, black bars; IL-7/HFD); Data are expressed as means ± SEM of 7 to 9 mice per group. ^#^ *p*<0.05, ^##^ *p*<0.01, ^###^ *p*<0.001, HFD *vs* SD within the same injection group; ^*^ *p*<0.05, ^**^ *p*<0.01, IL-7 injection *vs* PBS injection in the same diet group.

At that stage of our work, we hypothesized that IL-7 might impact on WAT mass increase and metabolic dysfunctions associated with the development of obesity, such as insulin and glucose intolerance. A protective role of IL-7 against obesity and insulin resistance has been suggested in IL-7-treated mice after pharmacological destruction of hypothalamus by monosodium glutamate [24] or gold thioglucose (IW, unpublished observation). Thus we assessed the consequences of a single subcutaneous injection of recombinant murine IL-7 in the more pathophysiological model of high-fat diet (HFD)-induced obesity. A unique administration of IL-7, at 2 weeks of diet, protected mice from HFD-induced body weight gain (Figure 3A). Food intake was not altered by IL-7 treatment (data not shown). A glucose tolerance test on overnight-fasted animals showed that, in contrast to PBS-treated HFD-fed (PBS/HFD) control mice, IL-7/HFD mice were not glucose intolerant after 5 weeks (Figure 3B) nor after 12 weeks (data not shown) of HFD feeding. This protective effect of IL-7 on HFD-induced glucose intolerance was also effective in condition of shorter duration of food removal (*i*.*e*. 6 hours over morning instead of overnight starvation period; data not shown). However, IL-7 injection had no effect on glycemia control in standard diet (SD)-fed animals. Moreover, the acute and early IL-7 injection protected mice from the HFD-induced increase of perigonadal WAT mass and, although less significantly, inguinal SCAT mass. Morphology of the brown adipose tissue (BAT) did not differ between the different experimental groups and its mass was not increased by the HFD feeding in the IL-7-treated mice (Figure 3C).

### Protective Effects of IL-7 Against HFD-induced Obesity are Associated with Decreased WAT Inflammation

One of the key features linking obesity and the development of insulin resistance is the chronic, low-grade inflammatory state of the WAT [28], [29] that is characterized by macrophage accumulation [30], [31] and activation [32], [33] which results in the secretion of various cytokines that eventually alter tissue insulin responsiveness. This critical process is preceded by the infiltration of lymphocytes which promote the recruitment and the activation of macrophages [34], [35]. Therefore, we compared the expression level of typical markers of immune cells and inflammatory mediators in the perigonadal WAT of mice from the different experimental groups of mice.

**Figure 4:**
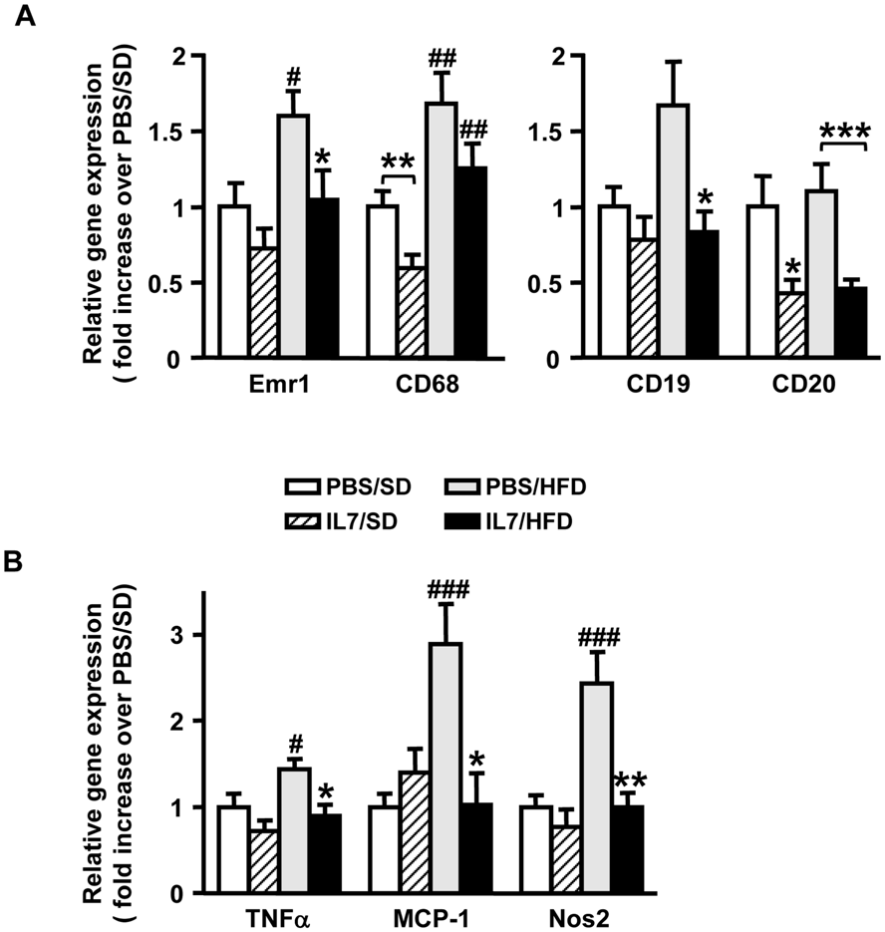
A single injection of IL-7 reduces WAT inflammation during C57BL/6 HFD feeding. Real-time quantitative PCR analysis of the perigonadal white adipose tissue from C57BL/6 mice after a unique injection with PBS or IL-7, 16 weeks after SD or HFD feeding. (**A**) Expression levels of macrophages (Emr1/F4-80, CD68) and B-cells (CD19, CD20) markers; and (**B**) expression levels of inflammatory markers (TNFα, MCP-1 and Nos2). Data are expressed as means ± SEM of 7 to 9 mice per group (white bars; PBS/SD, hatched bars; IL-7/SD, grey bars; PBS/HFD, black bars; IL-7/HFD). ^#^ *p*<0.05, ^##^ *p*<0.01, ^###^ *p*<0.001, HFD *vs* SD within the same injection group; ^*^ *p*<0.05, ^**^ *p*<0.01, ^***^ *p*<0.001, IL-7 injection *vs* PBS injection within the same diet group.

HFD feeding resulted in increased expression of macrophage markers Emr1 and CD68 (Figure 4A), TNFα, monocyte chemotactic protein-1 (MCP-1) and the activated-macrophage marker Nos2 (Figure 4B). The expression of all these markers was reduced in the WAT of IL-7-treated mice. The expression of the T-lymphocyte marker CD3ε or of the T-cell subset markers CD4 and CD8 was not altered in the WAT of PBS/HFD and IL-7/HFD mice after 16 weeks of diet (data not shown). The expression of the B-cell markers CD19 and CD20 were markedly decreased in the WAT of IL-7/HFD mice compared to PBS/HFD mice (Figure 4A). It is noticeable that in SD feeding conditions, IL-7 injection led to a significant decrease in CD68 and CD20 mRNA expression (markers specific for, respectively, macrophages and B-lymphocytes).

### A Single Administration of IL-7 Protects Immunodeficient SCID Mice from High-fat Diet-induced WAT Mass Increase but Not Against the Development of Glucose Intolerance and WAT Inflammation

To determine if the metabolic effects of IL-7 could be ascribed to any impact of the interleukin on lymphocytes, we then tested the consequences of acute IL-7 injection at the beginning of a HFD study in T- and B-cell deficient SCID mice.

**Figure 5:**
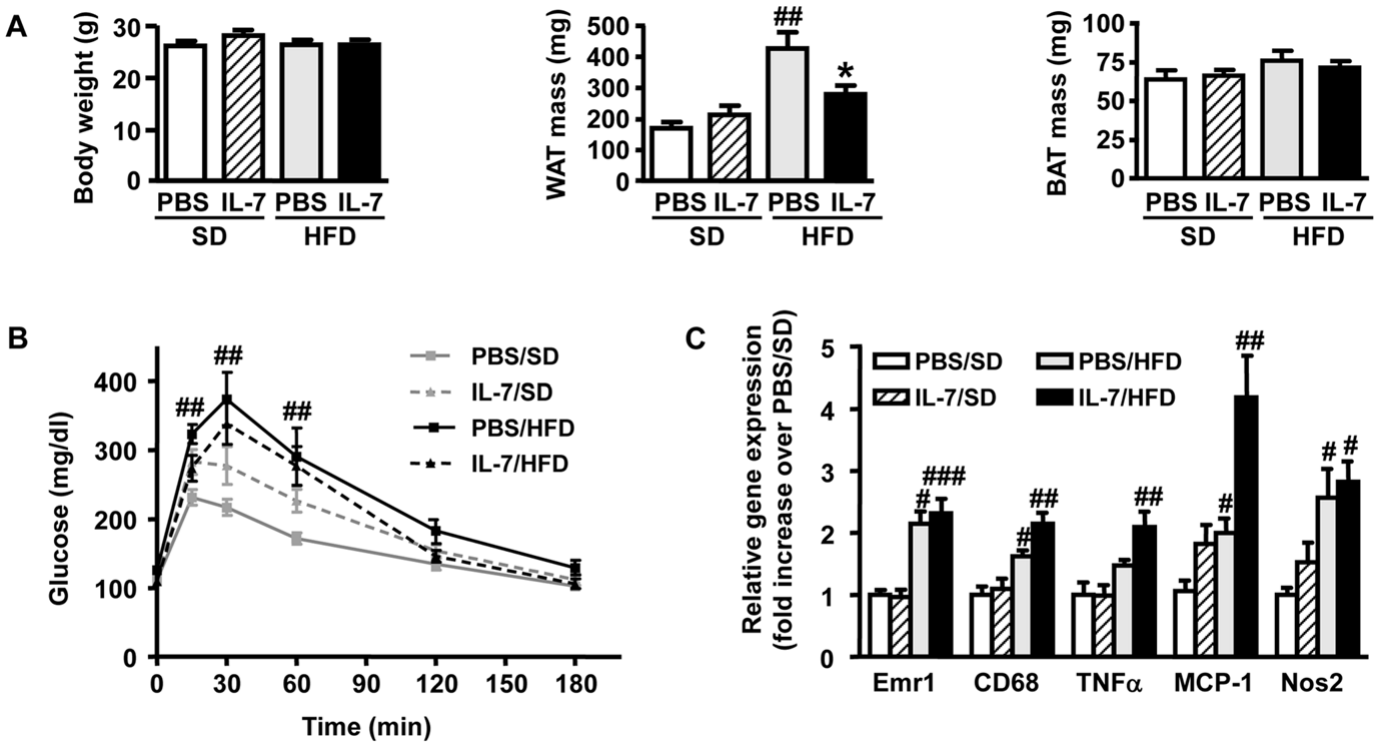
A single injection of IL-7 protects SCID mice from HFD-induced obesity but not from glucose intolerance and WAT inflammation. (**A**) Body weight and perigonadal WAT and BAT masses of SCID male mice fed during 15 weeks with SD or HFD (white bars; PBS/SD, hatched bars; IL-7/SD, grey bars; PBS/HFD, black bars; IL-7/HFD); (**B**) Intraperitoneal glucose tolerance test at 6 weeks of diet feeding (white bars; PBS/SD, hatched bars; IL-7/SD, grey bars; PBS/HFD, black bars; IL-7/HFD); (**C**) Expression levels of macrophage (Emr1/F4-80, CD68) and inflammation (TNFα, MCP-1, Nos2) markers in perigonadal WAT, after 15 weeks of diet feeding, using real-time quantitative PCR (white bars; PBS/SD, hatched bars; IL-7/SD, grey bars; PBS/HFD, black bars; IL-7/HFD). Data are expressed as means ± SEM of 8 PBS/SD mice, 6 PBS/IL-7 mice, 3 PBS/HFD mice and 9 IL-7/PBS mice. ^#^ *p*<0.05, ^##^ *p*<0.01, ^###^ *p*<0.001, HFD *vs* SD within the same injection group; ^*^ *p*<0.05, ^***^ *p*<0.001, IL-7 injection *vs* PBS injection in the same diet group.

As shown by Figure 5A, HFD feeding did not alter body weight in SCID mice, yet it led to a 2.5-fold increase in perigonadal fat mass. The administration of IL-7 in HFD-fed SCID mice significantly impaired this increase in WAT mass. The different experimental groups were similar regarding BAT mass and morphology.

In contrast, IL-7 injection did not protect anymore against HFD-induced glucose intolerance in lymphocyte-deficient SCID mice (Figure 5B). The same was true 9 weeks after injection (data not shown). Nevertheless, in SD feeding condition, IL-7-treated mice exhibited a transitory decrease in glucose clearance 4 weeks after the administration of IL-7, which was markedly improved later (data not shown). In addition, IL-7 had no effect on the perigonadal WAT expression of macrophage, as well as inflammatory, specific markers in lymphocyte-deficient HFD-fed SCID mice (Figure 5C).

## Discussion

Interleukin-7 (IL-7) is an immune cytokine that is critical for lymphocyte development and homeostasis [10], [11], [12], [13], [14], [15], [16], [17], [18], [19], recently found to be oversecreted by the visceral adipose tissue in obese subjects [9]. Yet, its potential role in white adipose tissue (WAT) development and metabolic functions has never been investigated. Here we describe an unexpected and dual role for IL-7 in WAT mass and insulin sensitivity control. In mice, constitutive IL-7 overexpression as well as IL-7 acute injection during high-fat diet (HFD) feeding reduced white adipose tissue mass. However, we show that the consequences of IL-7-induced WAT mass decrease on glucose homeostasis differ in the two models. Indeed, constitutive IL-7 over-expression leads to glucose intolerance and insulin resistance, traits that are commonly associated with lipodystrophy in both animals and humans [36]. In IL-7-treated HFD-fed wild-type animals, the reduced WAT mass is expectedly associated to protection against glucose intolerance that results from a decreased inflammation in WAT. Importantly, IL-7 protection against nutrient excess-induced glucose intolerance is lost in lymphocyte-deficient animals, suggesting that this protective effect of IL-7 evolves from a lymphocyte-dependent step of the inflammatory process that develops in the obese tissue.

Even though IL-7 constitutive overexpression is not comparable to IL-7 acute administration during nutritional excess both animal models present reduced adipose tissue mass. In addition, each model brings important clues onto the IL-7 impact in the control of glucose homeostasis, which may differ according to the developmental stage, nutritional status or sex. Regarding the latter, it is conceivable that gender difference between IL-7 overexpressing mice (females) and IL-7-injected animals (males) may participate to the reported effects on glucose homeostasis. Indeed, a sexual dimorphism has been described in mice that affected adipose tissue accumulation and gene expression as well as insulin sensitivity [37], [38], [39] and sex hormones such as estrogens have been convincingly reported to alter adipocyte biology and to impact on obesity-related co-morbidities, such as insulin resistance [40], [41], [42]. Nevertheless, even if estrous cycle, estrogen levels, or uterine weights have not been recorded in IL-7 transgenic female mice, no difference in food intake and/or meal size (all parameters that are controlled by estrogens, [43], [44], [45]) could be evidenced in these animals, compared to the corresponding control mice.

Our study mainly focused on the analysis of the perigonadal WAT, according to the well-recognized importance of this specific fat depot regarding lipolysis rate [46], preadipocyte/adipocyte cell dynamics [47] and inflammatory profile [48]. However, further investigation is definitely warranted to test whether regional (*i*.*e*. subcutaneous *versus* visceral) differences exist regarding adipose tissue responsiveness to IL-7.

IL-7 and IL-7Rα are highly expressed in the murine WAT and we show that, within the tissue, cells of the stromal vascular fraction (SVF) are likely the main IL-7 responsive cells, and not mature adipocytes. Indeed, while IL-7 mRNA is equally expressed in SVF cells and in mature adipocytes, as initially reported for the human adipose tissue [9], the specific IL-7Rα chain is mainly expressed in the SVF whilst less detectable in mature adipocytes.

Among the various cell-types that composed the SVF [49], [50], adipocyte progenitors could be IL-7 responder cells. Indeed, in the transgenic mouse model, IL-7 is overexpressed in the WAT (data not shown), and this sustained exposure of the tissue to IL-7 impacts on adipocyte cell-size. This suggests that IL-7 overexpression impairs the adipocyte differentiation process, thereby contributing to the development of insulin resistance in lipodystrophy-like transgenic animals. To support this hypothesis our preliminary data show that the expression of genes related to adipogenesis (such as PPARγ, C/EBPα and SREBP-1c) is decreased in the WAT of transgenic mice (data not shown). This is consistent with the report of decreased adipocyte differentiation in the adipose tissue of patients with HIV-related lipodystrophy, in association with insulin resistance [51]. It has been reported that IL-7 is produced by adipocyte progenitors and that its expression decreases during adipogenesis [9]. One hypothesis could be that the constitutive overexpression of IL-7 might have favoured the emergence and/or maintenance of a population of immature adipose cells with altered lipid storage and release capacities, explaining both the reduced adipocyte cell-size and the altered plasma lipid profile of Tg IL-7 mice.

Beside adipocyte progenitors, the SVF also contains immune cells that increasingly appear to play a key role in the induction and progression of the adipose tissue inflammatory state which contributes to the onset of insulin resistance that occurs in obesity [30], [31], [32], [33], [34], [35], [52], [53], [54]. We confirm that high-fat diet is associated with increased expression of macrophage specific markers in the adipose tissue and show that a single and early injection of IL-7 is sufficient to durably impede this HFD-induced effect, consequently protecting mice against glucose intolerance. The fact that acute IL-7 injection does not impact on any of these parameters in SD-fed mice supports that a pro-inflammatory and/or energy imbalance, such as the one triggered by obesity, is necessary for IL-7 to fully exert its regulatory effect.

The results we obtained in the immunodeficient HFD-SCID model bring some further clues on how IL-7 protects towards glucose intolerance in a context of obesity. Indeed, if IL-7-treated HFD-fed SCID mice still present a significant reduction in WAT mass, the IL-7 injection was inefficient to maintain glucose homeostasis and to prevent macrophage recruitment and activation, as reflected by the analysis of macrophage specific markers. This leads to conclude that, whereas the effects of IL-7 on WAT mass are lymphocyte-independent, its protective role against HFD-induced glucose intolerance requires the presence of functional lymphocytes. Since we show that IL-7 decreases the expression of B-lymphocyte specific surface markers in the WAT of HFD-fed immunocompetent mice, this cell-type might be proposed as an immune relay of the IL-7 effects on WAT inflammation. In contrast to macrophages and T-cells, the role of B-lymphocytes in the development of inflammation and insulin resistance in WAT is largely unknown, although these cells are recruited to adipose tissue shortly after the initiation of HFD (*e*.*g*. by 4 weeks) [55] and were recently reported to promote insulin resistance in mice [56]. Therefore, one could propose that IL-7, by decreasing the recruitment of these pathogenic B-cells in the adipose tissue, will limit macrophage recruitment/activation and thus, inflammation, consequently leading to the observed protective effect against glucose intolerance. In lymphocyte-deficient SCID mice, this IL-7-mediated effect on B-cells is lacking, leading to uncontrolled macrophage infiltration and loss of IL-7 protective effect against glucose intolerance.

Collectively, our findings indicate that the immune cytokine IL-7 could participate to the development of metabolic diseases through the modulation of the white adipose tissue. More specifically, we show here that IL-7 can play a dual role in the regulation of WAT mass and function. First, IL-7 regulates WAT mass in a lymphocyte-independent manner. How alteration of the adipogenesis, lipogenesis or lipolysis processes and/or increased oxidative capacity of the WAT concur to IL-7 effect on WAT mass remains to be established. Secondly, in condition of nutrient excess such as high-fat diet feeding, IL-7 protects durably against inflammation, and thus glucose intolerance, possibly by regulating the recruitment of immune cells - the canonical IL-7 responsive cell-types [10], [11], [12], [13], [14], [15], [16] - to the inflamed adipose tissue.

Several clinical trials using IL-7 are currently underway in cancer, HIV, HBV and HCV infections to remediate disease-associated immune deficits and our results in mice suggest that increasing IL-7 levels may also affect fat mass development and insulin sensitivity in the IL-7-treated patients. Moreover, our results reinforce the contribution of immune cells in insulin resistance in which IL-7 may play some important role. It shall help developing novel immune-based diagnostic and therapeutic modalities for managing white adipose tissue disorders such as obesity, lipodystrophy and insulin-resistance.

## Materials and Methods

### Animals

Heterozygous IL-7 transgenic mice on a C57BL/6J background expressed murine IL-7 under the control of the human keratin K14 promoter (Tg IL-7; [25]). Female wild-type littermate mice (WT) were used as controls. Body mass index was calculated for 8 week-old Tg IL-7 and WT mice. For acute IL-7 administration, we used C57BL/6J male mice (Janvier Laboratory, Le Genest-St-Isle, France) or C.B-17 SCID (Pasteur Institute, Lille, France) male mice.

### Diet-induced Obesity and Acute IL-7 Injection

Seven-week-old C57BL/6J and SCID male mice were fed either with a standard diet (AO4, SAFE, Augy, France) or with a high-fat diet (HFD 35.8% (wt/wt) fat from lard, Purified Diet 230HF, SAFE) containing, respectively, 5% or 60.6% in kcal of fat.

After 2 weeks of acclimatization to the diets, C57BL/6J and SCID mice were once subcutaneously injected with PBS or with recombinant murine IL-7 (0.3 µg/mouse, Peprotech, Neuilly-sur-Seine, France). Food intake and body weight were measured weekly, up to 16 weeks of diet.

### Hyperinsulinaemic-euglycaemic Clamp Experiment

Food was removed 5–6 hours before the *in vivo* protocol on catheterized animals. Tg IL-7 and WT mice underwent a 120 min-hyperinsulinaemic-euglycaemic clamp study with a prime continuous infusion of human insulin (Novonordisk, Copenhagen, Denmark) at a rate of 15 pmol/kg/min to raise plasma insulinaemia to ∼800 pM. Glucose (20%) was infused at variable rates to maintain euglycaemia. Insulin-stimulated whole-body glucose flux was estimated using a prime continuous infusion of HPLC-purified [3-^3^H] glucose (Amersham PerkinElmer, Waltham, MA, USA), throughout the clamp procedure. To estimate insulin-stimulated glucose-transport activity and metabolism in skeletal muscles, brown and white adipose tissues (subcutaneous (*i*.*e*. inguinal), and intraabdominal (*i*.*e*. perigonadal)), 2-deoxy-D[1-^14^C]-glucose (2DG; Amersham PerkinElmer) was administered as a bolus (37×10^4^ Bq) 45 min before the end of clamp procedure. In separate experiments, the basal glucose turnover rates were measured by continuous infusing of [3-^3^H] glucose (Amersham PerkinElmer) (74×10^3^ Bq/min) for 120 min, and plasma [^3^H] glucose concentration was determined every 10-min during the last 30 min.

### Intraperitoneal Glucose and Insulin Tolerance Tests

Glucose tolerance tests (GTT) were performed on overnight fasted mice injected i.p. with D-glucose (2 g/kg body weight, Sigma-Aldrich) [57], [58]. Glucose levels were measured by tail-tip bleeding with an automatic glucometer (ACCU-CHEK Performa, Roche) immediately before and 15, 30, 60, 180 and 360 min after the glucose injection. For determination of insulin sensitivity (ITT), mice were fasted for 4 h, and injected i.p. with porcine insulin (Sigma-Aldrich) (0.75 IU/kg body weight). Blood glucose levels were measured before and 15, 30, 45, 60, and 75 min after insulin injection.

### Gene Expression Analysis

One microgram of RNA extracted from the white adipose tissue was treated with Deoxyribonuclease I Amplification grade (Sigma), before being reverse-transcribed using Verso cDNA Kit and oligo-dT according to the manufacturer’s instructions (Thermo Scientific, ABgene, Epsom, UK). Real-time quantitative PCR was performed on 7900HT Fast real-Time PCR system using SYBR green chemistry (Applied Biosystems). Primers were designed using the software Primer Express 1.5 (Applied Biosystems) and primer sequences are available on request. GAPDH was used as an internal control to normalize gene expression. Results are expressed as fold-change compared to the PBS/SD group of the analyzed cohort.

### Characterization of IL-7 and IL-7R mRNA Expression in Insulin-sensitive Tissues

C57BL/6J male mice (Janvier Laboratory) fed with a standard diet, were sacrificed at the age of 8 weeks. Liver, skeletal muscles (*soleus* and *gastrocnemius*), hypothalamus and thymus were rapidly dissected and frozen in liquid nitrogen. Adipocytes and cells of the stromal vascular fraction (SVF) were isolated from perigonadal and subcutaneous white adipose tissues after digestion with collagenase (0.3 U/ml, Serva, Heidelberg, Germany) in DMEM supplemented with 2% BSA (Sigma). Following a 45 min digestion, cells were filtered through a 100 µm mesh filter and centrifuged at 600 g for 8 min. Floating adipocytes were then isolated from the SVF-cell fraction. Both fractions were washed twice in DMEM-2% BSA before being frozen in liquid nitrogen. Total RNA were extracted, reverse-transcribed and analyzed using real-time quantitative PCR as described above.

### Determination of Triglyceride Contents

Perigonadal adipose tissue was stored at −20°C in PBS containing 20 mM EDTA and 0.1% Triton X-100. Frozen samples were sonicated in 1 ml chloroform/methanol (2∶1 vol) and centrifuged so that the chloroform layer could be removed and the upper phase re-extracted. Pooled upper-phases were dried at 40°C and solubilized in isopropanol (HPLC Grade; Sigma-Aldrich, Lyon, France) for triglyceride content measurement (Sigma-Aldrich). DNA content was determined in the aqueous phase using the diaminobenzoic acid technique, by spectrophotometry.

### Adipose Tissue Morphometric Analysis

Perigonadal adipose tissues were fixed, embedded in paraffin, sectioned and stained with hematoxylin and eosin. Tissue sections images were acquired with a Zeiss Axiovert 100 M microscope. A minimum of 100 non-overlapping images was automatically acquired by serial scanning. Morphometric analyses were performed using the Image J software (NIH image, National Center for Biotechnology Information).

### Ethics Statement

Transgenic mice, WT littermate controls, C57BL/6J and C.B-17 SCID mice were bred and housed in specific pathogenic-free environment in Pasteur Institute’s animal facilities. All animals were maintained in a temperature-controlled (20±2°C) facility room with a strict 12-h light/dark and were given free access to regular food and water, unless otherwise stated.

Breeding, housing and experimentations were carried out according to the “Principles of laboratory animal care (NIH publication no. 85-23, revised 1985; http://grants1.nih.gov/grants/olaw/references/phspol.htm) as well as to the French and European guidelines of laboratory animal care (European Communities Council Directive of 1986, 86/609/EEC) and approved by the Departmental Direction of Veterinary Services (Prefecture of Lille, France; authorization number: 59-350152).

### Statistical Analyses

Data are presented as means ± SEM. The statistical significance of comparison between the different experimental groups was determined using two-way ANOVA test and the Mann-Whitney nonparametric test (GraphPad Prism software). *p* values less than 0.05 were considered statistically significant.

## Supporting Information

Table S1 **Metabolic parameters in IL-7 transgenic mice and wild-type controls.**
(TIF)
