# Supplementary figures and images for: Interleukin-7 Regulates Adipose Tissue Mass and Insulin Sensitivity in High-Fat Diet-Fed Mice through Lymphocyte-Dependent and Independent Mechanisms
Source: PLoS One. 2012 Jun 29;7(6):e40351. doi: 10.1371/journal.pone.0040351 (PMC3386973; doi:10.1371/journal.pone.0040351)

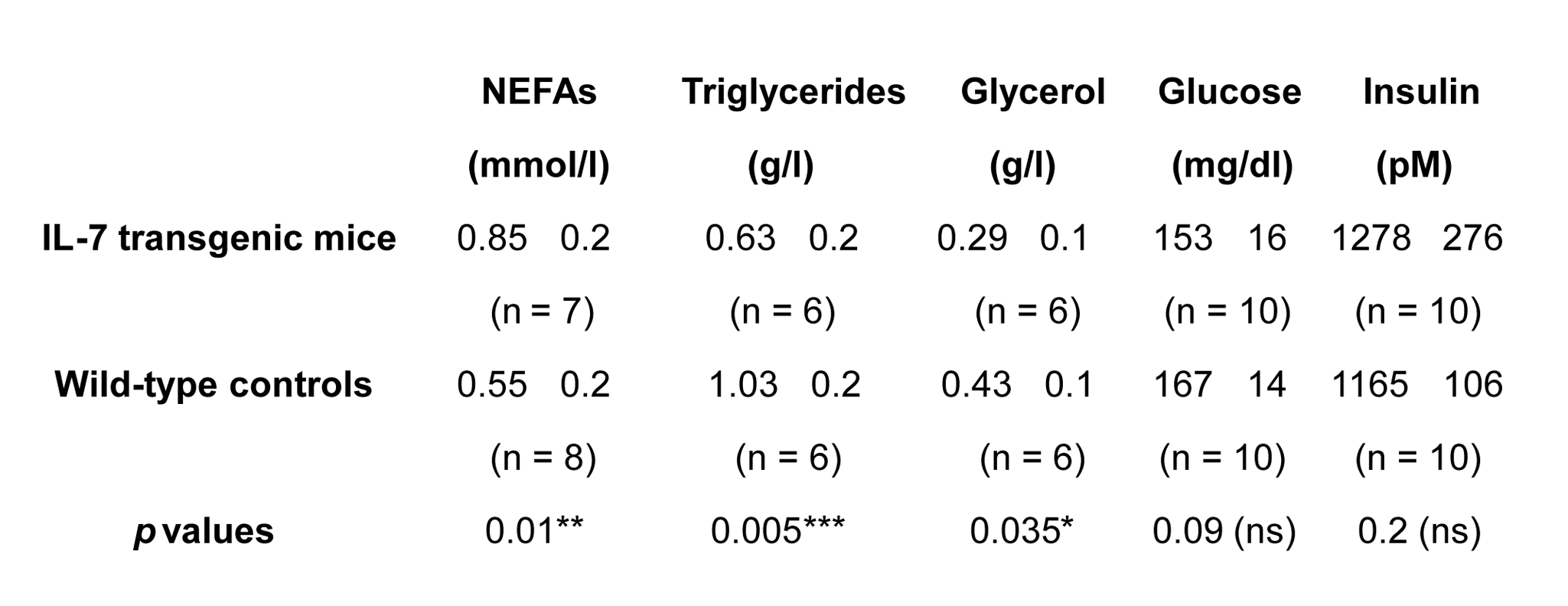

Supplement: Table S1 — Metabolic parameters in IL-7 transgenic mice and wild-type controls. (TIF) [file pone.0040351.s001.tif]
